# Highly Textured N-Type SnSe Polycrystals with Enhanced Thermoelectric Performance

**DOI:** 10.34133/2019/9253132

**Published:** 2019-11-11

**Authors:** Peng-Peng Shang, Jinfeng Dong, Jun Pei, Fu-Hua Sun, Yu Pan, Huaichao Tang, Bo-Ping Zhang, Li-Dong Zhao, Jing-Feng Li

**Affiliations:** ^1^State Key Laboratory of New Ceramics and Fine Processing, School of Materials Science and Engineering, Tsinghua University, Beijing 100084, China; ^2^College of Chemistry and Material Science, Shandong Agricultural University, Tai'an 271018, China; ^3^The Beijing Municipal Key Laboratory of New Energy Materials and Technologies, School of Materials Science and Engineering, University of Science and Technology Beijing, Beijing 100083, China; ^4^School of Materials Science and Engineering, Beihang University, Beijing 100191, China

## Abstract

Thermoelectric materials, which directly convert heat into electricity based on the Seebeck effects, have long been investigated for use in semiconductor refrigeration or waste heat recovery. Among them, SnSe has attracted significant attention due to its promising performance in both p-type and n-type crystals; in particular, a higher out-of-plane ZT value could be achieved in n-type SnSe due to its 3D charge and 2D phonon transports. In this work, the thermoelectric transport properties of n-type polycrystalline SnSe were investigated with an emphasis on the out-of-plane transport through producing textural microstructure. The textures were fabricated using mechanical alloying and repeated spark plasma sintering (SPS), as a kind of hot pressing, aimed at producing strong anisotropic transports in n-type polycrystalline SnSe as that in crystalline SnSe. Results show that the lowest thermal conductivity of 0.36 Wm^−1^ K^−1^ was obtained at 783 K in perpendicular to texture direction. Interestingly, the electrical transport properties are less anisotropic and even nearly isotropic, and the power factors reach 681.3 *μ*Wm^−1^ K^−2^ at 783 K along both parallel and perpendicular directions. The combination of large isotropic power factor and low anisotropic thermal conductivity leads to a maximum ZT of 1.5 at 783 K. The high performance elucidates the outstanding electrical and thermal transport behaviors in n-type polycrystalline SnSe, and a higher thermoelectric performance can be expected with future optimizing texture in n-type polycrystalline SnSe.

## 1. Introduction

Thermoelectric technology can convert thermal energy to electricity directly, which is a promising environmentally friendly power generation technology [1–4]. The conversion efficiency of thermoelectric technology is determined by the dimensionless figure of merit ZT = *S*^2^*σT*/*κ* for given thermoelectric materials, where *S*, *σ*, *κ*, and *T* are the Seebeck coefficient, electrical conductivity, thermal conductivity, and absolute temperature in Kelvin, respectively [5, 6]. In past decades, various efforts have been made to enhance thermoelectric performance and ZT records have been continuously renewed in thermoelectric community [7–16]. Recently, the binary compound SnSe has received considerable attention due to its promising thermoelectric performance in p-type crystals [17–19]. Subsequently, n-type SnSe crystals show a higher ZT value in the *a*-axis direction (out-of-plane), which is not originally expected and different from the high performance obtained along the *b*-axis direction (in-plane) in p-type SnSe crystals [20]. Well-established achievements in SnSe crystals indeed triggered enormous enthusiasm in the field of thermoelectrics; however, the time-cost method for crystal growth and its poor mechanical properties in crystals motivate us to investigate the polycrystalline SnSe. It is highly expected to get both the high performance and mechanical properties in textured polycrystalline SnSe with orientation grains [21]. Wang et al. prepared the textured polycrystalline SnSe via zone melting and hot pressing [22]. It was found that the high texturing degree is essential for obtaining high carrier mobility and therefore high thermoelectric performance; a peak ZT of 1.3 at 793 K was observed in the plane perpendicular to the hot pressing direction. Repeated pressurized sintering is more facile and effective to form the textured microstructure in some thermoelectric materials with a layered structure [23–25]. Moreover, the texturing processing with using mechanical alloying (MA) and spark plasma sintering (SPS) instead of conventional hot pressing will produce lower thermal conductivity because the rapid SPS process inhibits the grain growth for MAed fine powders [26, 27].

In this work, the thermoelectric properties of n-type polycrystalline SnSe were firstly realized through optimizing carrier concentration via Br doping. Then, it was found that a maximum ZT of ~1.3 can be obtained at 783 K in the sample with highly textured structures produced by MA and SPS technology. With further compensating Sn vacancies for the textured sample with high performance, the maximum ZT was boosted to ~1.5 at 783 K in n-type polycrystalline Sn_1.005_Se_0.94_Br_0.06_, This work indicates that the SnSe is one promising thermoelectric material and the thermoelectric performance for the materials with a layered structure could be effectively improved through utilizing its anisotropic transport behavior.

## 2. Results

**Figure 1:**
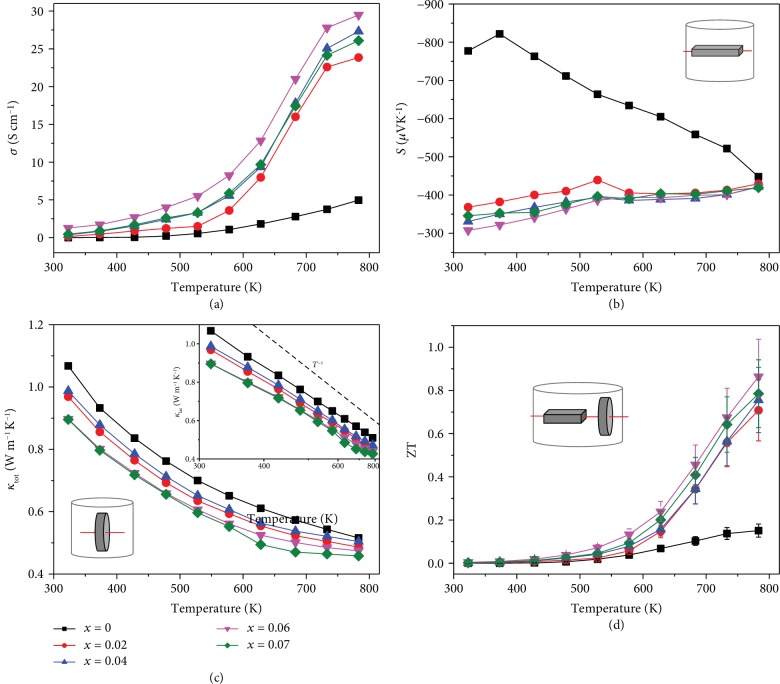
The temperature dependence of thermoelectric properties for SnSe_1-*x*_Br*_x_* (*x* = 0~0.07) along the direction that is perpendicular to the SPS pressure: (a) electrical conductivity, (b) Seebeck coefficient, (c) thermal conductivity (the inset shows the lattice thermal conductivity with a reciprocal coordinate of temperature), and (d) ZT values. The inset schematic figures shows the measurement directions for electrical and thermal transport properties.

First of all, the optimal amount of Br-doping in SnSe samples was determined. The temperature dependence of thermoelectric properties for SnSe_1-*x*_Br*_x_* (*x* = 0~0.07) along the perpendicular direction to the SPS pressure is shown in Figure 1. Undoped SnSe sample is an n-type semiconductor due to the Sn compensation, which has a low electrical conductivity and a large absolute value of Seebeck coefficient (see Figures 1(a) and 1(b)). Pristine SnSe is most often p-type due to Sn vacancies [17, 28]. Herein, trace Sn compensation (additional 0.1% Sn) makes the undoped SnSe exhibit n-type semiconductor characteristics because ball milling is more effective than a melting process to impede the generation of cation vacancies [29]. Br-doping obviously enhances the electrical conductivity, especially in the high temperature range, from 4.9 S cm^−1^ of the undoped sample to 29.5 S cm^−1^ of the *x* = 0.06 sample at 783 K. The absolute value of Seebeck coefficient and electrical conductivity in all Br-doped samples increase with the increasing temperature. This simultaneous enhancement in electrical conductivity and Seebeck coefficient was also reported on some p-type and n-type SnSe [30–34], indicating the nondegenerate characteristic of SnSe. Figure 1(c) shows the temperature dependence of the total and lattice thermal conductivity for SnSe_1-*x*_Br*_x_* (*x* = 0~0.07) samples along the direction that is perpendicular to the SPS pressure. Due to the low electronic thermal conductivity, the value of total thermal conductivity is similar to that of the lattice thermal conductivity, which decreases with the increasing Br-doping amount for the enhanced phonon scattering induced by defects in the lattice and/or at the grain boundaries. The inset in Figure 1(c) shows a *T*^−1^ relationship between *κ*_lat_ and temperature reveals that the Umklapp scattering dominates the phonon transport process. Along the perpendicular direction to the SPS pressure, the maximum ZT value of 0.9 is obtained for the *x* = 0.06 sample at 783 K, as shown in Figure 1(d). Based on the aforementioned optimal Br-doping amount, the influence of the SPSed texturing structure on the thermoelectric performance was subsequently studied.

**Figure 2:**
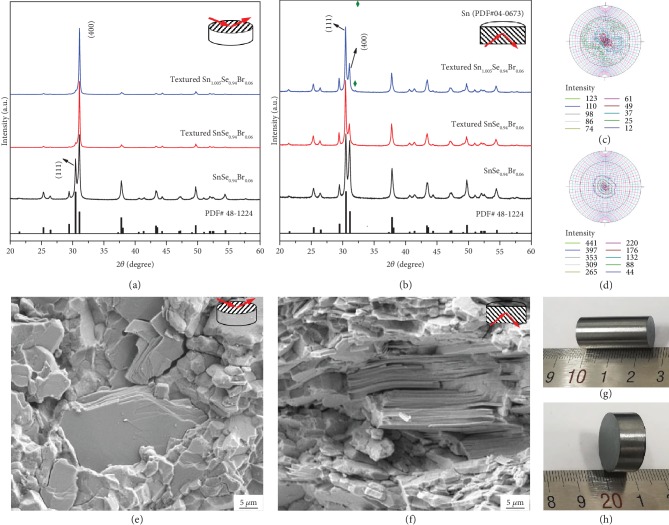
XRD patterns for the SnSe polycrystalline samples before and after texturing process along the directions that are perpendicular (a) and parallel (b) to the SPS pressure; the standard pattern of SnSe (PDF# 48-1224) is also plotted for comparison. (c, d) (111) and (400) pole figures of textured SnSe_0.94_Br_0.06_ along the direction that is perpendicular to the SPS pressure. (e, f) SEM images of the fractured surfaces for textured Sn_1.005_Se_0.94_Br_0.06_ along the two directions. (g) and (h) are the typical samples for as-SPSed and SPS texturing 3 times, respectively.

Figures 2(a) and 2(b) present the XRD patterns for the SnSe polycrystalline samples before and after the texturing process along the directions that are perpendicular and parallel to the SPS pressure, respectively. The peaks show a well match to the standard pattern of SnSe (PDF# 48-1224) and can be indexed based on the orthorhombic phase (space group Pnma). The variation of (111) and (400) peak intensities for the SnSe_0.94_Br_0.06_ indicates the anisotropic structure along the different directions, and this anisotropy is significantly enhanced by SPS pressing, as shown by the fact that the (111) peak almost disappears and (400) peak obviously weakens along the perpendicular and parallel direction in the textured SnSe_0.94_Br_0.06_ and Sn_1.005_Se_0.94_Br_0.06_, respectively. Furthermore, in the textured Sn_1.005_Se_0.94_Br_0.06_, Sn metal phase (PDF# 04-0673) is observed and its effect on the thermoelectric performance will be discussed later. The texturing degree is then determined by the orientation factors, *F*, using the following equations [35]:

(1) $$\begin{array}{c} F=\frac{P-P_{0}}{1-P_{0}}, \end{array}$$

(2) $$\begin{array}{c} P_{0}=\frac{I_{0}\left(h00\right)}{\sum I_{0}\left(hkl\right)}, \end{array}$$

(3) $$\begin{array}{c} P=\frac{I\left(h00\right)}{\sum I\left(hkl\right)}, \end{array}$$

where *P* and *P*_0_ are the ratio of integrated intensities of all (*h*00) planes to the intensities of all (*hkl*) planes for preferentially and randomly oriented samples. The *F* values of the SnSe_0.94_Br_0.06_, textured SnSe_0.94_Br_0.06_, and Sn_1.005_Se_0.94_Br_0.06_ are 0.18, 0.66, and 0.71, respectively, where the standard diffraction pattern of SnSe was used as a reference. In order to understand the anisotropy more intuitively, the pole figures of two representative crystallographic directions for textured SnSe_0.94_Br_0.06_ along the perpendicular direction to the SPS pressure are given in Figures 2(c) and 2(d). It is clear that the discrete spots of (400) pole are very concentrated compared to that of (111) pole, which is consistent with the sharp (400) peak in Figure 2(a), being indicative of the significantly oriented texture. Moreover, the textured morphologies are also clearly illustrated in the SEM images as shown in Figures 2(e) and 2(f). Corresponding to the layered crystal structure along the parallel direction to the SPS pressure, the flake grains appear along the perpendicular direction, indicating that all crystallites roughly orient in a similar direction. Two typical samples used in this work are shown in Figure 2(g) for one-time SPS and Figure 2(h) for texturing treatment with 3-time SPS. Both samples have the metallic luster, and an even denser microstructure could be obtained after the texturing process as the relative densities increase from ~92% to >97% of the theoretical density of SnSe.

**Figure 3:**
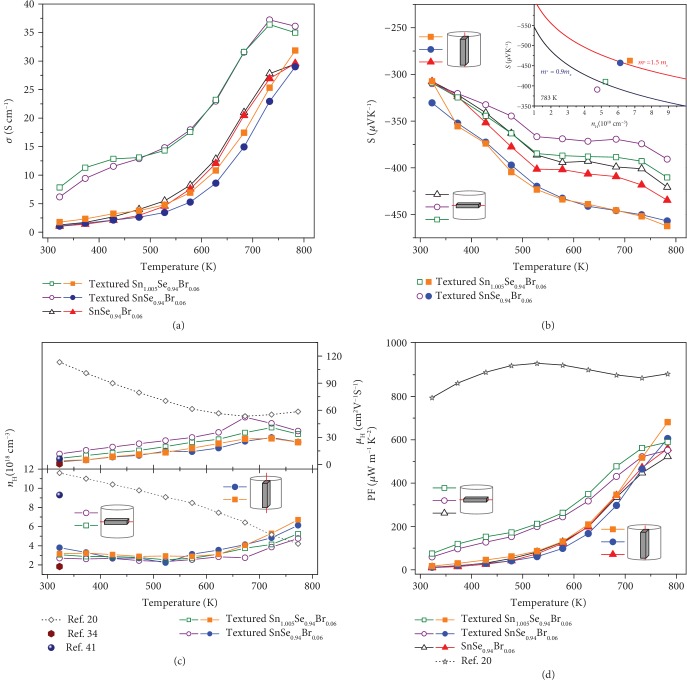
The temperature dependence of the electrical transport properties for the polycrystalline SnSe samples before and after texturing process; unfilled symbols and filled symbols present the properties measured along the directions that are perpendicular and parallel to the SPS pressure, respectively: (a) electrical conductivity, (b) Seebeck coefficient, (c) carrier mobility and carrier concentration, and (d) power factor; reported data [20, 34, 41] are also plotted for comparison; the inset shows the Pisarenko relationship; Seebeck coefficient as a function of carrier concentration, with different effective masses. The inset schematic figures show the measurement directions for electrical and thermal transport properties.

The temperature dependence of the electrical conductivity for the polycrystalline SnSe samples before and after texturing process is investigated and shown in Figure 3(a); unfilled and filled symbols present the measurement results along the directions that are perpendicular and parallel to the SPS pressure, respectively. Compared with the SnSe_0.94_Br_0.06_, textured SnSe_0.94_Br_0.06_ and Sn_1.005_Se_0.94_Br_0.06_ have the slightly lower electrical conductivity in the parallel direction (cross-layer) to the SPS pressure, while they exhibit better electrical performance along the perpendicular direction (in-the-layer). Such a converse impact comes from the alignment of grains during the texturing process, leading to the attenuation of grain boundary scattering in the layer. The measured carrier mobility, as shown in Figure 3(c), confirms the above explanation as the carrier mobility increases largely in the perpendicular direction with a similar carrier concentration. It should be noted that the carrier mobility exhibits an increased tendency with increasing temperature before ~700 K, which clarifies that the enhancements of electrical conductivity with temperature actually originate from the increase of mobility rather than the activation of carriers. This phenomenon is uncommon in semiconductors and related to the grain boundary scattering of carriers [36], which is also detected in Mg_3_Sb_2_ [37]. The increased mobility at elevated temperature realizes the simultaneous optimization of electrical conductivity and Seebeck coefficient, leading to a superior power factor at 783 K, as shown in Figure 3(d).

Furthermore, the Seebeck coefficient also shows an interesting trend corresponding to the SPSed texturing structure. Compared to the similar value of the Seebeck coefficient in the two directions for the SnSe_0.94_Br_0.06_, as shown in Figure 3(b); the absolute value of the Seebeck coefficient increases in the parallel direction to the SPS pressure, but decreases in the perpendicular direction after the texturing process. In order to have a deep insight into the variation of the Seebeck coefficient, the carrier concentration dependence of the Seebeck coefficient at 783 K, usually known as Pisarenko lines [38], is presented in the inset of Figure 3(b). The single parabolic band (SPB) model with acoustic phonon scattering is applied to roughly determine the density of state (DOS) effective mass. A DOS effective mass of 0.9 *m*_e_ can match the data along the perpendicular direction to the SPS pressure for the textured samples, while the Seebeck coefficient along the parallel direction falls onto the line with an effective mass of 1.5 *m*_e_. In our opinion, the anisotropy of the Seebeck coefficient and DOS effective mass are caused by the strong anisotropic nature due to the SPSed texturing structure, which may affect the transport directions of the carriers in crystals. The dependence of the Seebeck coefficient on the crystallographic orientation is also reported in *β*-FeSi_2_ crystals and BiSb thin films [39, 40]; nevertheless, we need to admit that the mechanism between strong textures and the DOS effective mass in polycrystalline SnSe is still not clear and needs further study.

**Figure 4:**
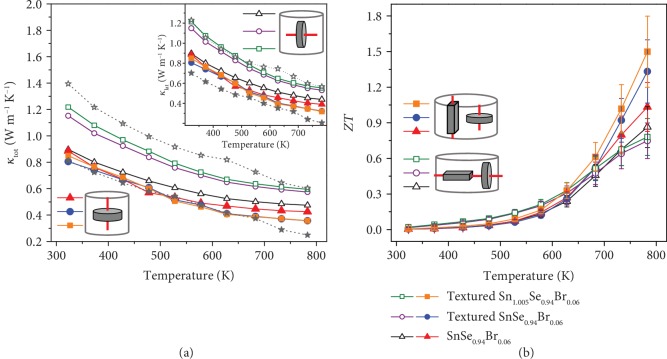
The temperature dependence of (a) thermal conductivity (unfilled and filled star symbols present the reported data along the in-plane and out-of-plane directions in the n-type SnSe crystal [20], respectively; the inset shows lattice thermal conductivity) and (b) ZT values for the polycrystalline SnSe samples before and after texturing process; unfilled symbols and filled symbols present the properties measured along the directions that are perpendicular and parallel to the SPS pressure, respectively. The inset schematic figures show the measurement directions for electrical and thermal transport properties.

Figure 4(a) shows the temperature dependence of the total thermal conductivity (with an inset of the lattice thermal conductivity) for the polycrystalline SnSe samples before and after the texturing process; unfilled symbols and filled symbols present the properties measured along the directions that are perpendicular and parallel to the SPS pressure, respectively. The reported thermal conductivities along the in-plane (unfilled star symbols) and out-of-plane (filled star symbols) directions in the n-type SnSe crystal are also given for comparison [20]. An enhanced textured structure elevates the thermal conductivity in the perpendicular direction due to the increased relative density and the weakened grain boundary scattering, as shown by unfilled symbols in Figure 4(a). Fortunately, as shown by filled symbols in Figure 4(a), the strong texture-induced violent cross-layer scattering significantly decreases the thermal conductivity along the parallel direction to the SPS pressure, which is consistent with the fact that the intrinsically ultralow thermal conductivity of SnSe comes from the layered structure [41]. The lowest thermal conductivity of 0.36 Wm^−1^ K^−1^ at 783 K is obtained in the textured SnSe_0.94_Br_0.06_. Although this thermal conductivity is still larger than the reported value of ~0.25 Wm^−1^ K^−1^ in n-type crystalline SnSe and polycrystalline SnSe bulks [20, 42], the increased electrical transport properties lead to a high ZT value of 1.3 at 783 K for the textured SnSe_0.94_Br_0.06_, as shown in Figure 4(b), whose better performance lines in the direction that is parallel to the SPS pressure. In summary, the texturing process increases the ZT value in the cross-layer direction, suggesting the effectiveness of texture modulation on improving the thermoelectric property of n-type SnSe polycrystalline bulks.

**Figure 5:**
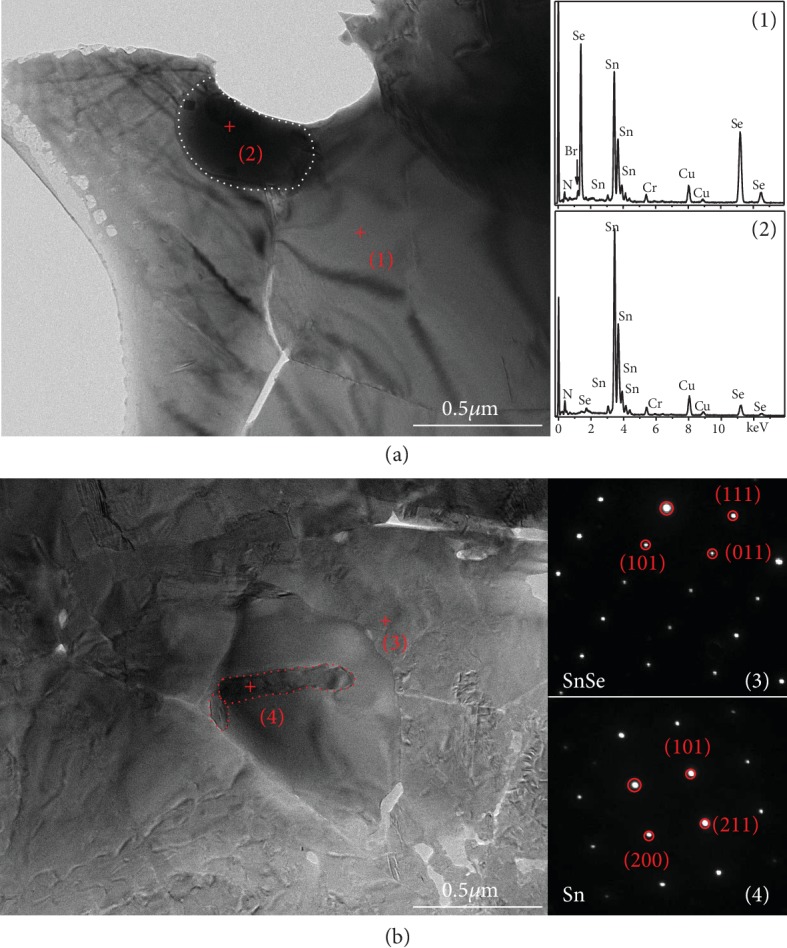
TEM images of textured Sn_1.005_Se_0.94_Br_0.06_ along the directions that are perpendicular (a) and parallel (b) to the SPS pressure. The EDS and SAED patterns are taken from the red cross region (1) (2) and (3) (4) in (a) and (b), respectively; the inset shows the existence of Sn cobblestone-shaped grains (circled by white dotted line) and bar-like grains (circled by red dotted line) in the SnSe-based matrix.

To further enhance the thermoelectric properties of SPSed texturing Br-doped SnSe samples, the introduction of excess Sn is expected to promote the electrical transport properties as donor doping. As shown in Figures 3(a) and 3(b), it is as expected that the Sn-excessed samples have slightly higher electrical conductivity and a similar absolute value of the Seebeck coefficient compared to other samples. Thus, a 12% enhancement of power factor was realized in the parallel direction of textured Sn_1.005_Se_0.94_Br_0.06_ (681.3 *μ*Wm^−1^ K^−2^ at 783 K), which is shown in Figure 3(d). The Sn element phase is detected and shown in Figure 2, and the TEM images in Figures 5(a) and 5(b) also illustrate the presence of Sn particles with different morphologies for the textured Sn_1.005_Se_0.94_Br_0.06_ along the perpendicular and parallel directions to the SPS pressure, respectively.  Figure 5(a) shows the cobblestone-shaped grains with a size of 300~400 nm in the layer, and the EDS clearly clarifies the SnSe matrix (red cross (1)) and Sn particle (red cross (2) and circled by a white dotted line). In the cross-layer, a lot of pleat-shaped grains are observed and the SAED pattern of the red cross (3) in Figure 5(b) is indexed as SnSe of the Pnma space group. Furthermore, some bar-like grains circled by a red dotted line, as shown in SAED pattern of the red cross (4), which are determined as Sn with the I41/amd (141) space group. These Sn particles with the width of ~100 nm lies in the grain boundary or embeds into the SnSe grains and would usually increase the thermal conductivity of SnSe-based thermoelectric materials due to its intrinsically high thermal conductivity. However, as shown in Figure 4(a), the Sn-excessed sample has the very similar total thermal conductivity as the textured SnSe_0.94_Br_0.06_. The inset in Figure 4(a) indicates that the lattice thermal conductivity mainly contributes to the total thermal conductivity, and it is also anisotropic and decreases significantly along the parallel direction to the SPS pressure due to the enhanced phonons scattering in the cross-layer. Interestingly, the lattice thermal conductivity of textured Sn_1.005_Se_0.94_Br_0.06_ is slightly lower than that of textured SnSe_0.94_Br_0.06_ even with the existence of Sn metal phase, reaching 0.32 Wm^−1^ K^−1^ at 783 K. The effective inhibition of phonon transport may be attributed to the strengthened phonon scattering, which is induced by the bar-like Sn particles in the SnSe matrix. Benefiting for the enhanced electrical performance and maintenance of low lattice thermal conductivity, the largest ZT value of ~1.5 at 783 K is obtained in SPSed texturing Sn_1.005_Se_0.94_Br_0.06_, as shown in Figure 4(b).

## 3. Discussion

This work demonstrated the performance enhancement in n-type polycrystalline SnSe alloys through optimizing carrier concentration and designing textured microstructures. Textured SnSe_0.94_Br_0.06_ was fabricated by MA and repeated SPS process, and the ZT values at 783 K steeply increased from 1.0 to 1.3. Excess Sn was used in the textured Sn_1.005_Se_0.94_Br_0.06_ to enhance the electrical transport properties, as shown in Figure 3(c); the carrier concentration is similar in the two directions, and it is slightly larger than that of n-type SnSe crystal (star symbol) at ~783 K [20]. However, significant anisotropy of the carrier mobility was observed, and the value of ~25.0 cm^2^ V^−1^S^−1^ (783 K) in the cross-layer direction (filled symbols) is far less than that of the SnSe crystal (star symbol), as shown in Figure 3(c), in which overlapping charge density fills the interlayers and facilitates the high out-of-plane carrier mobility [20]. The degradation of cross-layer carrier mobility in the present textured samples is mainly caused by the interlayer scattering due to the enhanced polycrystalline texture structures.

The texturing process makes the ZT values show significant anisotropy along the different directions to the SPS pressure, as shown in Figure 4(b), and greatly benefitted the thermoelectric performance in the parallel cross-layer direction which was similar to the enhancement of out-of-plane ZT in n-type SnSe crystals due to 3D charge transport behavior from the overlapped charge density between the SnSe layers. A maximum cross-layer ZT value of ~1.5 was achieved at 783 K in the textured Sn_1.005_Se_0.94_Br_0.06_, but this value is still much smaller than that in n-type SnSe crystals. Except for the low carrier mobility in the cross-layer direction, another reason is the high thermal conductivity. Although the low thermal conductivity of 0.36 Wm^−1^ K^−1^ (783 K) for textured samples is reduced ~17% compared to SnSe_0.94_Br_0.06_ along the parallel direction (filled symbols) to the SPS pressure, as shown in Figure 4(a), it is still higher than the out-of-plane thermal conductivity of the SnSe crystal [20]. On the other hand, compared to the in-plane thermal conductivity (unfilled star symbols), the lower values for textured samples prove the effect of grain boundary scattering on reducing thermal conductivity. This indicates that although the texturing process makes the high orientation factors of 0.71, leading to enhanced grain boundary scattering in the cross-layer direction, it is not as good as the phonon scattering by the complete orientation in SnSe crystals. So further aligning the crystals in the low thermal conductivity along the cross-layer direction by the texturing process is important to enhance the thermoelectric performance. Additionally, the surface oxides on polycrystalline SnSe materials during the polycrystal preparation will also increase the thermal conductivity and simultaneously deteriorate carrier mobility [43, 44]. Based on the new strategies provided in this work, further strengthening the textured structures and improving the cross-layer electron transport through enhancing carrier concentrations will help us to enhance the thermoelectric performance of the materials with strong anisotropic structures.

## 4. Materials and Methods

### 4.1. Sample Synthesis

High-purity Sn (powder, 99.99%), Se (powder, 99.99%), and SnBr_2_ (powder, 99.4%) were weighed in a glove box with an Ar atmosphere. Different amounts of raw materials were mixed according to the stoichiometric ratio of Sn*_y_*Se_1-*x*_Br*_x_* (*x* = 0~0.07, *y* = 1, and 1.005) in an Argon-protective atmosphere (>99.5%) and then subject to mechanical alloying on a planetary ball milling machine (QM-3SP4, Nanjing University, China) at 450 rpm for 15 hours. Stainless steel vessels and balls were used, with a weight ratio of ball to powder of 20 : 1. The mechanically alloyed powders were densified by SPS (SPS-211Lx, Japan) in a *Φ*12 mm graphite mold at 793 K for 5 min under an axial pressure of 50 MPa in vacuum. Considering that undoped SnSe is always p-type due to cation vacancies, an additional 0.1% Sn (unmarked in the formula) was added for compensation in SnSe_1-*x*_Br*_x_* (*x* = 0~0.07) in order to obtain n-type samples.

The texturing process can be described as follows: The polished cylinder-shaped samples of *Φ*12 were put into a *Φ*15 mm graphite mold and SPSed again to obtain textured bulks through the extra space. The As-synthesized bulks were then polished and followed by the third-time SPS in *Φ*20 mm graphite mold to obtain textured bulks through the extra space, finally resulting in cylinder-shaped samples with a thickness of 9 mm. The sintering process of the second- and the third-time SPS is exactly the same with that of the first-time SPS. In order to express expediently in the text, the samples after the texturing process (3-time SPS) were named textured samples, e.g., textured SnSe_0.94_Br_0.06_, distinguished to the SnSe_0.94_Br_0.06_ without textures.

### 4.2. Phase and Microstructure Identification

Samples' phase purity and textured structures were studied using X-ray diffraction (XRD, RINT 2000, Rigaku, Japan) with Cu K*α* radiation. X-ray pole figures were measured to study the crystal direction distribution using a Bragg-Brentano focusing with 2 mm Schulz slit and 0.67° divergence slit, 1.3 mm anti-scatter slit, and 0.6 mm receiving slit (XRD, D/max-RB, Rigaku, Japan). Scanning electron microscopy (SEM) images were taken in the secondary electron (SE2) and backscattering electron (BSE) detector modes using a field emission scanning electron microscope (FE-SEM, JSM-7001, JEOL, Japan). Microstructure and selected area electron diffraction (SAED) as well as the energy-dispersive X-ray spectroscopy (EDS) patterns were observed using a transmission electron microscope (TEM, JEM-2100, JEOL, Japan). Copper rings (AZS21, EMD, China) and glue (ELEMER, USA) were used to hold the samples for TEM, and so elements Cr and N existing in there were detected in the EDS spectra, as shown in Figure 5.

### 4.3. Thermoelectric Property Measurement

The sintered samples were cut perpendicularly and parallelly to the SPS pressure direction into bars with dimensions about 9 mm × 2 mm × 2 mm for the simultaneous measurement of the Seebeck coefficient (*S*) and electrical conductivity (*σ*) using the Seebeck coefficient/electric resistance measuring system (ZEM-3, Ulvac-Riko, Japan) under a helium atmosphere from 323 K to 783 K. Heating and cooling cycles gave repeatable electrical properties, which indicate that the properties are thermally stable. The electrical properties obtained from different slices cut from the same pellets were similar along the same pressing direction, attesting the homogeneity of the samples. Disk-shaped samples were cut from the sintered samples along the perpendicular and parallel directions to the SPS pressure, respectively. After polishing and coating with a thin layer of graphite to minimize errors from the emissivity of the material, the disk-shaped samples (*Φ*6 mm × 1 mm) were used to measure the thermal diffusivity (*D*) with the laser flash diffusivity method (Netzsch, LFA 457, Germany) ([Supplementary-material supplementary-material-1]). The value of specific heat (*Cp*) was referenced from Ref. [20]. The density (*d*) was got from the Archimedes method ([Supplementary-material supplementary-material-1]). Finally, the total thermal conductivity was calculated via the equation *κ* = *DC*_p_*d*. The magnitude of electrical thermal conductivity *κ*_ele_ could be calculated by the Wiedemann-Franz equation, *κ*_ele_ = *LσT*, where *L* is the Lorenz number, *σ* is the electrical conductivity, and *T* is the absolute temperature. The calculated *L* is shown in [Supplementary-material supplementary-material-1]. Lattice thermal conductivity (*κ*_lat_) is calculated by subtracting electronic thermal conductivity (*κ*_ele_) from total thermal conductivity (*κ*_tot_). The Hall coefficient (*R*_H_) of samples was measured under a reversible magnetic field of 0.52 T (ResiTest 8340DC, Toyo, Japan). Carrier concentration and carrier mobility were calculated by *n*_H_ = 1/(*eR*_H_) and *μ*_H_ = *R*_H_*σ*, respectively. Due to the layered structure of SnSe, both the Hall coefficient and the electrical and thermal transport properties of the samples were measured along the same directions. The overall uncertainty of ZT is about 20% due to 5% uncertainty of the Seebeck coefficient, electrical conductivity, and thermal conductivity, respectively.

## Supplementary Materials

Supplementary Materials Figure S1: thermal diffusivity (a) and Lorenz number (b) as a function of temperature for SnSe1-xBrx (*x* = 0~0.07) and textured SnySe0.94Br0.06 (*y* = 1 and 1.005). The symbols || and ⊥ represent the measurement directions that are parallel and perpendicular to the SPS pressing direction, respectively. Table S1: measured sample density of SnSe1-xBrx (*x* = 0~0.07) and textured SnySe0.94Br0.06 (*y* = 1 and 1.005).

## References

[1] Bell L. E. (2008). Cooling, heating, generating power, and recovering waste heat with thermoelectric systems. *Science*.

[2] Snyder G. J., Toberer E. S. (2008). Complex thermoelectric materials. *Nature Materials*.

[3] Zhao L. D., Dravid V. P., Kanatzidis M. G. (2014). The panoscopic approach to high performance thermoelectrics. *Energy & Environmental Science*.

[4] Li J. F., Liu W. S., Zhao L. D., Zhou M. (2010). High-performance nanostructured thermoelectric materials. *NPG Asia Materials*.

[5] Borup K. A., de Boor J., Wang H. (2015). Measuring thermoelectric transport properties of materials. *Energy & Environmental Science*.

[6] Zhang X., Zhao L. D. (2015). Thermoelectric materials: energy conversion between heat and electricity. *Journal of Materiomics*.

[7] Zhao L. D., Hao S., Lo S. H. (2013). High thermoelectric performance via hierarchical compositionally alloyed nanostructures. *Journal of the American Chemical Society*.

[8] Shi X., Yang J., Salvador J. R. (2011). Multiple-filled skutterudites: high thermoelectric figure of merit through separately optimizing electrical and thermal transports. *Journal of the American Chemical Society*.

[9] Uher C. (2001). Chapter 5 skutterudites: prospective novel thermoelectrics. *Semiconductors and Semimetals*.

[10] Zhao L. D., He J., Berardan D. (2014). BiCuSeO oxyselenides: new promising thermoelectric materials. *Energy & Environmental Science*.

[11] Dresselhaus M. S., Chen G., Tang M. Y. (2007). New directions for low-dimensional thermoelectric materials. *Advanced Materials*.

[12] Heremans J. P., Jovovic V., Toberer E. S. (2008). Enhancement of thermoelectric efficiency in PbTe by distortion of the electronic density of states. *Science*.

[13] Pei Y. Z., Shi X., LaLonde A., Wang H., Chen L., Snyder G. J. (2011). Convergence of electronic bands for high performance bulk thermoelectrics. *Nature*.

[14] Biswas K., He J., Blum I. D. (2012). High-performance bulk thermoelectrics with all-scale hierarchical architectures. *Nature*.

[15] Tan G. J., Shi F. Y., Hao S. Q. (2016). Non-equilibrium processing leads to record high thermoelectric figure of merit in PbTe–SrTe. *Nature Communications*.

[16] Kim S. I., Lee K. H., Mun H. A. (2015). Dense dislocation arrays embedded in grain boundaries for high-performance bulk thermoelectrics. *Science*.

[17] Zhao L. D., Lo S. H., Zhang Y. (2014). Ultralow thermal conductivity and high thermoelectric figure of merit in SnSe crystals. *Nature*.

[18] Zhao L. D., Tan G. J., Hao S. (2016). Ultrahigh power factor and thermoelectric performance in hole-doped single-crystal SnSe. *Science*.

[19] Wei T. R., Wu C. F., Li F., Li J. F. (2018). Low-cost and environmentally benign selenides as promising thermoelectric materials. *Journal of Materiomics*.

[20] Chang C., Wu M. H., He D. S. (2018). 3D charge and 2D phonon transports leading to high out-of-plane *ZT* in n-type SnSe crystals. *Science*.

[21] Hong M., Chen Z. G., Yang L. (2017). Enhancing the thermoelectric performance of SnSe_1−*x*_Te*_x_* nanoplates through band engineering. *Journal of Materials Chemistry A*.

[22] Wang X., Xu J., Liu G. Q. (2017). Texturing degree boosts thermoelectric performance of silver-doped polycrystalline SnSe. *NPG Asia Materials*.

[23] Zhao L. D., Zhang B. P., Li J. F., Zhang H. L., Liu W. S. (2008). Enhanced thermoelectric and mechanical properties in textured n-type Bi_2_Te_3_ prepared by spark plasma sintering. *Solid State Sciences*.

[24] Noudem J. G. (2009). A new process for lamellar texturing of thermoelectric Ca_3_Co_4_O_9_ oxides by spark plasma sintering. *Journal of the European Ceramic Society*.

[25] Sui J. H., Li J., He J. Q. (2013). Texturation boosts the thermoelectric performance of BiCuSeO oxyselenides. *Energy & Environmental Science*.

[26] Pan Y., Li J. F. (2016). Thermoelectric performance enhancement in n-type Bi_2_(TeSe)_3_ alloys owing to nanoscale inhomogeneity combined with a spark plasma-textured microstructure. *NPG Asia Materials*.

[27] Chen Z. G., Shi X. L., Zhao L. D., Zou J. (2018). High-performance SnSe thermoelectric materials: progress and future challenge. *Progress in Materials Science*.

[28] Maier H., Daniel D. R. (1977). SnSe single crystals: sublimation growth, deviation from stoichiometry and electrical properties. *Journal of Electronic Materials*.

[29] Dong J. F., Sun F. H., Tang H. C. (2019). Medium-temperature thermoelectric GeTe: vacancy suppression and band structure engineering leading to high performance. *Energy & Environmental Science*.

[30] Wei T. R., Wu C. F., Zhang X. (2015). Thermoelectric transport properties of pristine and Na-doped SnSe_1−*x*_Te*_x_* polycrystals. *Physical Chemistry Chemical Physics*.

[31] Ge Z. H., Song D. S., Chong X. Y. (2017). Boosting the thermoelectric performance of (Na,K)-codoped polycrystalline SnSe by synergistic tailoring of the band structure and atomic-scale defect phonon scattering. *Journal of the American Chemical Society*.

[32] Zhang Q., Chere E. K., Sun J. (2015). Studies on thermoelectric properties of n-type polycrystalline SnSe_1‐*x*_S*_x_* by iodine doping. *Advanced Energy Materials*.

[33] Ge Z. H., Qiu Y. Q., Chen Y. X. (2019). Multipoint defect synergy realizing the excellent thermoelectric performance of n-type polycrystalline SnSe via Re doping. *Advanced Functional Materials*.

[34] Chang C., Tan Q., Pei Y. L. (2016). Raising thermoelectric performance of n-type SnSe via Br doping and Pb alloying. *RSC Advances*.

[35] Lotgering F. K. (1959). Topotactical reactions with ferrimagnetic oxides having hexagonal crystal structures—I. *Journal of Inorganic and Nuclear Chemistry*.

[36] Wei T. R., Tan G. J., Zhang X. M. (2016). Distinct impact of alkali-ion doping on electrical transport properties of thermoelectric p-type polycrystalline SnSe. *Journal of the American Chemical Society*.

[37] Kuo J. J., Kang S. D., Imasato K. (2018). Grain boundary dominated charge transport in Mg_3_Sb_2_-based compounds. *Energy & Environmental Science*.

[38] Leng H. Q., Zhou M., Zhao J., Han Y. M., Li L. F. (2016). The thermoelectric performance of anisotropic SnSe doped with Na. *RSC Advances*.

[39] Takeda M., Kuramitsu M., Yoshio M. (2004). Anisotropic Seebeck coefficient in *β*-FeSi_2_ single crystal. *Thin Solid Films*.

[40] Cho S., Kim Y., DiVenere A., Wong G. K., Ketterson J. B., Meyer J. R. Anisotropic Seebeck and magneto-Seebeck coefficients of Bi and BiSb alloy thin films.

[41] Li C. W., Hong J., May A. F. (2015). Orbitally driven giant phonon anharmonicity in SnSe. *Nature Physics*.

[42] Li S., Wang Y. M., Chen C. (2018). Heavy doping by bromine to improve the thermoelectric properties of n-type polycrystalline SnSe. *Advanced Science*.

[43] Lee Y. K., Luo Z., Cho S. P., Kanatzidis M. G., Chung I. (2019). Surface oxide removal for polycrystalline SnSe reveals near-single-crystal thermoelectric performance. *Joule*.

[44] Banik A., Biswas K. (2019). A game-changing strategy in SnSe thermoelectrics. *Joule*.

