# Supplementary material for: Highly Textured N-Type SnSe Polycrystals with Enhanced Thermoelectric Performance
Source: Research (Wash D C). 2019 Nov 11;2019:9253132. doi: 10.34133/2019/9253132 (PMC6946256; doi:10.34133/2019/9253132)
Supplement: Supplementary Materials — Figure S1: thermal diffusivity (a) and Lorenz number (b) as a function of temperature for SnSe1-xBrx (x = 0~0.07) and textured SnySe0.94Br0.06 (y = 1 and 1.005). The symbols || and ⊥ represent the measurement directions that are parallel and perpendicular to the SPS pressing direction, respectively. Table S1: measured sample density of SnSe1-xBrx (x = 0~0.07) and textured SnySe0.94Br0.06 (y = 1 and 1.005). [file 9253132.f1.docx]

Supplementary Materials:

# Highly Textured N-type SnSe Polycrystals with Enhanced Thermoelectric Performance

**Peng-Peng Shang,^1,2^ Jinfeng Dong,^1^ Jun Pei,^3^ Fu-Hua Sun,^1^ Yu Pan,^1^ Huaichao Tang,^2^ Bo-Ping Zhang,^3^ Li-Dong Zhao,^4,*^ and Jing-Feng Li^1,*^**

^1^State Key Laboratory of New Ceramics and Fine Processing, School of Materials Science and Engineering, Tsinghua University, Beijing 100084, China

*^2^College of Chemistry and Material Science, Shandong Agricultural University, Tai’an 271018, China*

*^3^The Beijing Municipal Key Laboratory of New Energy Materials and Technologies, School of Materials Science and Engineering, University of Science and Technology Beijing, Beijing 100083, China*

*^4^School of Materials Science and Engineering, Beihang University, Beijing 100191, China*

^*^Correspondence to: zhaolidong@buaa.edu.cn; jingfeng@mail.tsinghua.edu.cn


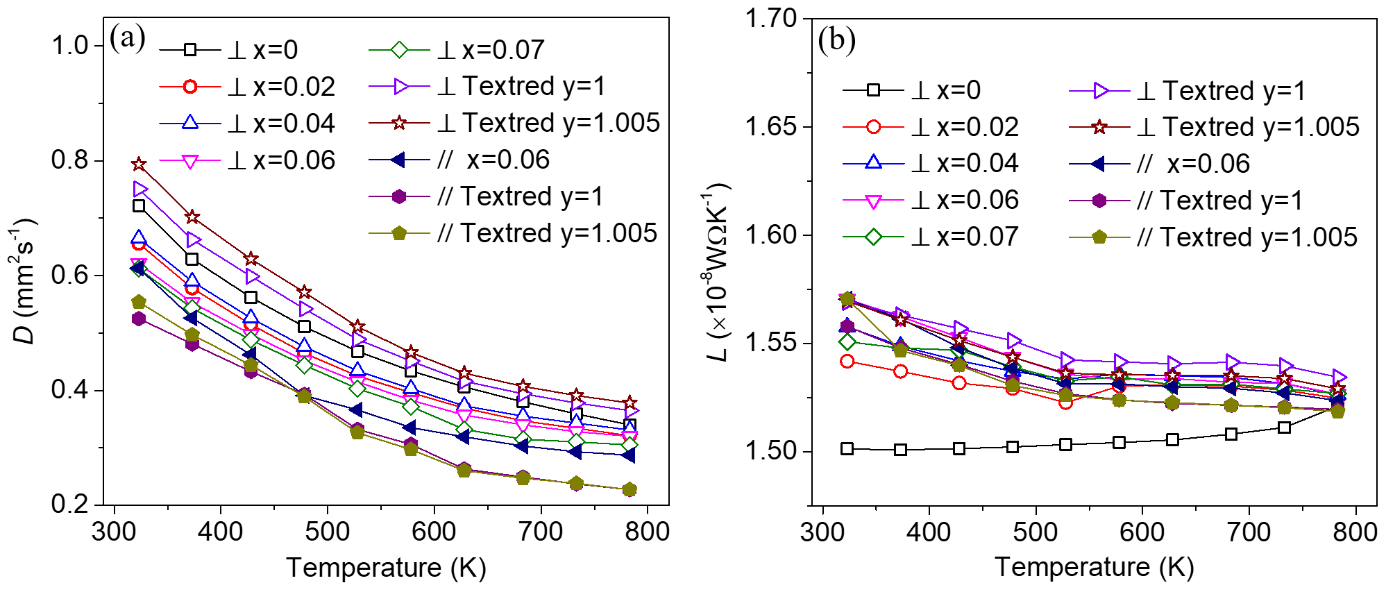


Figure S1: Thermal diffusivity (a) and Lorenz number (b) as a function of temperature for SnSe_1-_*_x_*Br*_x_* (*x* = 0 ~ 0.07) and textured Sn*_y_*Se_0.94_Br_0.06_ (*y* = 1 and 1.005). The symbol ∥ and ⊥ represent the measurement directions that are parallel and perpendicular to the SPS pressurre, respectively.

Table S1: Measured sample density of SnSe_1-_*_x_*Br*_x_* (*x* = 0 ~ 0.07) and textured Sn*_y_*Se_0.94_Br_0.06_ (*y* = 1 and 1.005).

| Sample | Density [g cm^-3^] |
| --- | --- |
| *x*= 0 | 5.86 |
| *x*= 0.02 | 5.85 |
| *x*= 0.04 | 5.88 |
| *x*= 0.06 | 5.73 |
| *x*= 0.07 | 5.80 |
| Textured *y* = 1 | 6.07 |
| Textured *y* = 1.005 | 6.09 |
